# Pollinator nutrition and its role in merging the dual objectives of pollinator health and optimal crop production

**DOI:** 10.1098/rstb.2021.0170

**Published:** 2022-06-20

**Authors:** Jeremy Jones, Romina Rader

**Affiliations:** School of Environmental and Rural Science, University of New England, Armidale, NSW, Australia

**Keywords:** pollination, crop, non-bee, pollinator, insect

## Abstract

Bee and non-bee insect pollinators play an integral role in the quantity and quality of production for many food crops, yet there is growing evidence that nutritional challenges to pollinators in agricultural landscapes are an important factor in the reduction of pollinator populations worldwide. Schemes to enhance crop pollinator health have historically focused on floral resource plantings aimed at increasing pollinator abundance and diversity by providing more foraging opportunities for bees. These efforts have demonstrated that improvements in bee diversity and abundance are achievable; however, goals of increasing crop pollination outcomes via these interventions are not consistently met. To support pollinator health and crop pollination outcomes in tandem, habitat enhancements must be tailored to meet the life-history needs of specific crop pollinators, including non-bees. This will require greater understanding of the nutritional demands of these taxa together with the supply of floral and non-floral food resources and how these interact in cropping environments. Understanding the mechanisms underlying crop pollination and pollinator health in unison across a range of taxa is clearly a win–win for industry and conservation, yet achievement of these goals will require new knowledge and novel, targeted methods.

This article is part of the theme issue ‘Natural processes influencing pollinator health: from chemistry to landscapes’.

## 1. Introduction

Insect pollinators are intricately linked to the production of globally important crops and the reproduction of wild plants [1,2] and provide critical services that enhance human welfare and maintain biodiversity. Growing concerns about declining pollinator populations in a time of increasing production of pollinator-dependant crops have highlighted the urgent need to understand the stressors faced by pollinators in agricultural landscapes [1,3]. The protection of remnant habitat and introduction of floral food resources on and around farms is thought to support bees, non-bees and other beneficial insects [4], yet these actions do not necessarily guarantee improved crop yields or increased pollinator health, even when they improve bee species diversity [5]. While most conservation actions have focused on bees, non-bee taxa are also significant pollinators [6], yet little is currently known about the resource needs of these taxa, both in regard to the optimal selection of plant species and the types of non-floral resources that would best support larval, reproductive and nesting needs [7].

Effective crop pollination services arise from the interactions between flower visitors and plants, resulting in increased yields and/or higher quality produce [1,8]. Many studies report increased floral-visitor abundances when providing additional non-crop floral resources [9], however, the addition of non-crop floral resources *per se* does not necessarily guarantee yields. This is because pollinators do not visit all flowers available, and differences in foraging behaviour can result in some flowers being used over others [10]. This in turn may negatively impact crop pollination via competition if neighbouring non-crop floral resources are used more than crop flowers, resulting in pollinator dilution [11]. Alternatively, the addition of non-crop floral resources may result in crop pollination facilitation by increasing pollinator food quantity and temporal availability, leading to greater pollinator abundance, but this can depend on plant species composition, landscape context and field configuration [9,12].

Pollinator health has been recently defined as a multilevel, spatio-temporally unconstrained measure of the wellbeing, resilience and ecological functionality of individuals through to populations of pollinators [13]. Providing additional non-crop floral resources is thought to support pollinator health by providing nectar and pollen for diet and reproductive needs [14,15]. However, little is known about which plants provide the best quality resources for particular taxa as rewards from different plant species vary in nutritional value [16], and some species appear to be detrimental to pollinator health owing to pathogen transmission [17]. Furthermore, bees may exhibit foraging preferences for food sources contaminated with neonicotinoids even when their health is compromised [18], and these compounds may mobilize from crop sites to floral strip plantings [19]. Understanding the mechanisms underlying crop pollination and pollinator health together is clearly a win–win for industry and conservation but will require additional data and novel, targeted methods.

## 2. Pollinators and optimal crop production

For insect pollinated crops, optimal crop production is closely linked to both the number of insect visits to flowers and the quality of these visits. Crops are pollinated by a wide diversity of insect taxa, including bees, flies, beetles and moths [6]. Any given pollinator taxon has a unique set of behavioural and morphological traits, which influence the crop species visited, rate at which they visit crop flowers and the amount of pollen deposited. The quality of deposited pollen is also important and can be influenced by cultivar-specific variation in pollen viability and pollen self-incompatibility, as many crops are obligate out-crossers [20]. At the field-scale, the abundance and identity of crop pollinators also matters. A high diversity of pollinators is thought to provide crop pollination resiliency [21], and synergistic effects can occur between pollinating taxa [22]; however, many crops are pollinated by a small number of dominant species [23]. High pollinator abundance can directly translate to increased flower visitation and yield [24], and in cropping systems, this is often achieved through the addition of managed taxa such as honeybees. However, achieving optimal pollination by increasing pollinator density alone may not translate to increased yields if the pollinators are ineffective pollinators of a target crop, prefer non-crop floral resources or perform poorly in the crop environment [11,24,25]. Conversely, extremely high visitation rates can cause damage to crop flowers with detrimental effects on yield [26]. Hence strategies to achieve optimal crop pollination will vary by crop identity, pollinator assemblage and the environment in which a crop is cultivated.

## 3. Agricultural impacts on pollinator health

Threats and challenges to pollinators have become increasingly well-known, driven heavily by habitat loss (and hence loss of suitable forage), pathogens and pesticide usage [27]. Agricultural landscapes are often associated with changes in pollinator community composition and reductions in pollinator abundances and body size, and these changes may have negative consequences for crop pollination outcomes [28,29]. One of the major factors affecting pollinators in agriculture landscapes is poor nutrition, driven by extensive monoculture plantings and loss of semi-natural habitat [30]. Negative feedbacks may occur between malnutrition and pathogens, exacerbating the stresses faced by pollinators [31].

Many efforts to improve pollinator abundance and health have been undertaken by governments, institutions and individual farmers [9,12]. These efforts have focused on providing habitat to support the nutritional and nesting requirements of pollinators through floral resource plantings. While overall these objectives have provided evidence that floral resource additions do support pollinator health in cropping environments [32], the effects on crop pollination service delivery have been mixed [9,12].

While most studies have focused on managed bees, knowledge gaps still exist in our understanding of how visitation rate to crop flowers, and ultimately yield, relates to colony strength and hive density, and surrounding co-flowering resource abundance. Studies on visitation rates to flowers suggest there is an optimal level of flower visitation needed for many crops, and at levels above or below this, crop production may be negatively impacted, either by insufficient or excessive visitation [26] or unnecessary investment in hired pollination services [24]. Colony stocking rate guidelines for crops also do not consider the farm-scale variations in the contributions by, and interactions with, wild pollinators [24], which in some cases can be high [33]. These factors probably have repercussions for both crop pollination service delivery as well as pollinator health in cropping environments and beyond. This is because managed taxa stocked too highly may result in inter-colony competition for food resources and greater horizontal pathogen transfer [34,35], with potential impacts on colony health, wild pollinator populations and surrounding plant community structure through altered pollination networks [34,36,37]. Migratory beekeeping, which itself can be detrimental to the health of managed bees [38], also raise the possibility for off-site effects extending beyond the crop site, for example, through pathogen spread to heterospecifics [39] or through pasturing depleted colonies in protected areas [40]. To secure crop pollination, pollinator health and conservation outcomes, it is critically important that these interactions are examined further altogether.

## 4. Crops as floral food resources

To enhance crop pollinator health, it is important to understand the nutritional needs of crop pollinators in tandem with the type and quality of foods accessed. Crop floral resources show phenotypic variations in their nutritional content, at both the inter- and intra-species levels [41]. At the plant level, spatial and temporal patterns may occur in floral resource quantity and quality. This may be owing to plant mating system and crop layout. For example, in the dioecious crop kiwifruit (*Actinidia deliciosa*), which lacks nectar, a small number of male plants are distributed across orchards and provide high protein pollen, yet the majority of plants are female with inviable pollen with lower protein quantity, creating a restricted nutritional environment [42]. Daily variations also occur in nectar secretion and pollen dehiscence across many crop types, and these may not overlap with the foraging periods of some taxa [43]. Compounding the phenotypic variation in nutritional quality across different crops and cultivars, plant environment [44], disease [45], florivores [46], agricultural inputs [47], floral microorganisms [48] and below-ground interactions [49] also alter the chemical profiles of crop floral resources. These environmental variations in floral resource quality can have positive or negative consequences on pollinator fitness and crop pollination [47,50,51] indicating that management strategies which support favourable floral nutritional profiles for crop pollinators may be possible, for example, by using fungal inoculants or altering fertilizer regimes. Field and landscape level crop and non-crop floral diversity and abundance shape the nutritional landscape for pollinators and these resources fluctuate over seasonal and annual cycles [52], highlighting the need for long-term monitoring in crop pollinator studies.

An evaluation of the nutritional value of a crop depends on the pollinator taxon in question. Specialist crop pollinators (e.g. the oligolectic squash bees, *Peponapis* spp.) may obtain most of their nutritional requirements from single crops, yet generalist pollinators (e.g. *Bombus* spp.) fed an exclusive diet of these crops may develop poorly [53]. In addition, crop floral resources may be protected by floral morphologies or deterrent compounds [53,54], limiting their exploitation by generalist foragers. These differences highlight the need for greater understanding of the nutritional value of crops to specific pollinators and for unique bioregional approaches to pollinator management. For example, supporting endemic oligolectic crop pollinators may require greater emphasis on meeting non-floral resource needs, yet beyond their biogeographical range, mismatches between the nutritional profiles of crops and the nutritional requirements of generalist crop pollinators may result in a greater need to provide alternative forage. However, even within the range of oligolectic crop pollinators, supporting generalist pollinators is often essential owing to altered flowering phenology of plants in cultivation, which may not overlap with the flight periods of oligolectic pollinators [55].

The most common floral resources provided by crop plants are pollen and nectar; however, crops may also offer floral resources in the form of eliasomes (e.g. acerola, *Malphigia emarginata* [56]), nutritious petals (e.g. feijoa, *Feijoa sellowiana* [57]) and brood sites (e.g. oil palm, *Elaeis* spp. [58]). Pollen contains proteins, free amino acids, lipids, sterols, vitamins, minerals and also low concentrations of carbohydrates [16]. While protein quantity is important, protein quality also matters and depends on the amount of essential amino acids present in the protein [59]. Crops may provide unsuitable ratios of essential amino acids or lipids; therefore, pollinators of these crops may require access to non-crop flowers to obtain adequate dietary requirements and may adjust their foraging accordingly [60]. Essential fatty acids and sterols are required by pollinators and must also be obtained from diet [61]. Nectar is primarily composed of water, sugars and low concentrations of amino acids [16]. Other trace components in nectar include minerals and secondary metabolites, and these substances can have medicinal, attractant or repellent effects on pollinators [54]. In addition to floral rewards, pollinators may consume a wide diversity of non-floral nutritional resources, which can be particularly important for non-bee reproductive and larval needs [6].

## 5. Nutritional resources: comparing and contrasting bees and non-bees

One major distinction between bee and non-bee pollinators is that bees commonly obtain most of their nutritional requirements from flowers, whereas non-bees may obtain nutrition from a variety of non-floral resources and, in contrast to bees (apart from some wasps), are not reliant on food provisioning by adults [61]. Pollen is the main source of proteins, lipids and sterols for growth, development and reproduction in bees. For non-bees that consume pollen, pollen can provide either essential or supplemental nutrition for reproduction and egg maturation [62]. For honeybees, essential amino acids necessary for energy, growth and reproduction have been documented, and comparisons between these requirements and the ratios found in many crops have driven estimations of the nutritional value of crops for this taxon [63]. This knowledge will be valuable for future studies in selecting non-crop floral resources that may complement the nutritional profiles of crops. Yet, for many important non-bee pollinators, knowledge is lacking not only in terms macronutrient requirements but also on which types of floral or non-floral resources are consumed.

Non-bee pollinators may consume nectar and/or pollen as larvae, adults or both [62]. For the most common generalist crop flower visitors—Diptera, Lepidoptera and Coleoptera, larval nutrition is primarily obtained from non-floral resources, while adults visit crop flowers for energy needs and/or to obtain nutrition for reproduction and egg development [6]. Many non-bee pollinators are able to use nutritional stores obtained as larvae for egg maturation [64] and therefore rely less on supplementary protein or lipids that may be obtained from pollen—these taxa visit flowers primarily for energy needs. Despite this, many taxa that can use nutrients acquired during larval development may still benefit from additional floral protein, carbohydrates and amino acids for subsequent egg cycles, as nutritional stores may become depleted after the first egg cycle [64]. Supporting these taxa will require understanding of how the quantity and quality of non-floral resources impact adult fecundity and which plant species provide suitable floral resources for adults needs.

## 6. Non-floral nutritional requirements of pollinators

In addition to pollen, nectar and floral oils, bees may consume honeydew, extrafloral nectar, mycelial exudates and mineral rich water [7]. For stingless bees, resins in cerumen may also be a component of honey and pot-pollen [65]. Non-bees may use many of these same resources but also require additional non-floral resources for mating, egg maturation and larval development. Evidence suggests that the nutritional value of these resources can vary, and this may impact the health of non-bee pollinators. For example, in Diptera that visit manure for adult protein needs, dung protein content which is influenced by livestock forage quality, can impact egg maturation [66,67]. Veterinary medicines used for livestock management may also contaminate manure, with off-target impacts on beneficial coprohilic insects including Diptera [68]. A mixed adult diet can also be important, as access to plant sugars in addition to animal protein increases adult longevity and decreases the time taken for ovary maturation in some Calliphoridae [69]. While data are broadly lacking on the role of non-floral resource quality for non-bee crop pollinators, it is to be expected that variations in these resources impact populations of these taxa and is an area requiring further research.

## 7. Merging optimal crop production and pollinator health

### (a) Floral resource management

Floral resource management is a key tool to support pollinator health and diversity. Plant species richness is closely linked to pollinator species richness and increasing floral diversity around farms can benefit pollinator abundance and diversity [32,70]. However, the identity and composition of plant species matters, as not all studies report positive correlations [32] and some plants may facilitate pathogen transmission [17].

Recent reviews suggest pollinator habitat enhancement does not consistently increase crop pollination outcomes [5,9,12]. This highlights the critical need to understand competitive and facilitative pollination interactions between crops and co-flowering plants, which may include other crops [71,72]. These interactions arise from the integration of crops into surrounding plant-pollinator networks, which shape crop pollination outcomes by influencing the behaviour, diversity and demography of shared pollinators [72]. Enhancing crop pollination outcomes therefore requires consideration of crops and surrounding vegetation communities together, and how these are best structured, managed and integrated to support pollinator health, ecosystem services and conservation goals.

Increasingly, efforts are being made to evaluate the nutritional value of specific plants in pollinator enhancement schemes, which can reveal unexpected patterns and assist in formulating better guidelines for species mixes that complement the nutritional profiles of crop flowers [73–75]. Visitation studies suggest that in some cases, relatively few sown species in habitat enhancement schemes are used by pollinators, highlighting the need for broader research into which plants are most appropriate [10,76]. Some key species in seed mixtures may be particularly important for bees, non-bee pollinators and crop pest predators [77,78]. Selecting plants that are exclusively used by one group of pollinators may allow targeting of habitat interventions to promote specific ecosystem functions or enhance conservation of taxa of concern [77]. To mitigate the risks of exotic plant invasions and support multifunctional habitat goals, native plants should be prioritized [78]; however, the value of native plants to pollinators can be influenced by source provenance [79] and consideration must also be given to the potential for some species to facilitate agricultural pests [80]. Trade-offs therefore exist when determining plant selection criteria, and these may also encompass economic and practical aspects of sourcing and establishing propagules, genetic diversity risks of using non-local provenance plants and limitations regarding available evidence to support their inclusion [80,81]. These trade-offs require much greater discussion, especially for biodiversity hotspots and grower-funded habitat enhancement.

Diverse forage provides opportunities for pollinators to achieve more balanced nutritional intakes and to obtain medicinal phytochemicals, which can enhance forager performance, colony health and population densities and reduce pathogen prevalence or transmission [82–85]. However, diversity alone may not be sufficient to satisfy the nutritional requirements of pollinators if those resources provide inadequate quantities of food resources or are otherwise unattractive or unavailable to be used by foraging crop pollinators [86,87]. In this way, floral abundance, composition and phenology are also important and have ramifications for species selection when the land available for habitat intervention may be limited.

Seasonal bottlenecks may exist in the quantity of nectar or pollen available in agricultural landscapes and this may impact social bee colony growth and provisioning, which may have long-term effects on a colony [88,89]. For solitary bees and non-bee crop pollinators, floral resource continuity is important, but its role requires further study [90–92]. Floral resource continuity may be enhanced by the availability of sequentially flowering crops [71] or through diverse floral resource plantings, although commercial pollinator seed mixes may not provide an adequate range of flowering phenology [93]. In tropical climates, planting non-crop plant species with steady-state flowering characteristics can increase bee species abundances around crop fields, but positive effects on crop pollination may be limited, especially when crop floral abundance is low [94]. This result highlights the need for further study towards understanding if tailoring plantings to provide floral continuity, but with reduced temporal overlap with crop flowering, can maximize crop pollination facilitation while mitigating the effects of co-flowering resource competition.

Intensive agricultural practices and landscape simplification impact pollinator abundance, a major driver of crop yields [23]. Mass-flowering crops can increase the abundance of some pollinators [95] and in some cases, facilitate pollination of co-flowering crops [71], yet can also negatively impact surrounding crops by increasing heterospecific pollen deposition and interfering with pollination [96]. Extensive crop fields and narrow flowering phenology means supporting sufficient abundances of wild pollinators to provide crop pollination services in mass-flowering systems remains a major challenge [3]. Strategies for re-designing cropping systems to mitigate pollinator declines while enhancing crop pollination include reducing field size, increasing habitat complexity and connectivity [97], and careful selection and configuration of pollinator-complementary crops [71]. Configuration of wildflower strips and semi-natural habitat can also impact crop pollination outcomes and fields may benefit from designs which integrate non-crop floral resources within field centres [98]. Placement of pollinator magnet plants may enhance pollination of less-attractive neighbouring plants in some circumstances [72], yet this remains to be demonstrated in cropping contexts. Adoption of crop pollinator enhancement strategies by stakeholders will require both clear outcomes and practical pathways that afford flexibility for growers, and in this regard, attention has to be paid to encouraging strategies that are also achievable for growers in the short term.

Crop pollinator habitat enhancement studies have primarily focused on hedgerows and rotational wildflower strips composed of commercially available annual and perennial forbs [12], the outcomes of which can vary depending on initial habitat establishment success [99]. Nitrogen enrichment in agricultural landscapes alters plant community structure to favour nitrophytic plants, which is a major challenge in ecosystem rehabilitation efforts [100]. Encroachment of grasses and other ruderal weeds can result in temporal declines in the value of these interventions for maintaining pollinator diversity and abundance [101], yet few studies have examined the effects of pollinator habitat enhancement on crop pollination beyond four years since establishment [9], largely precluding inference about the possible role of planted trees. Flowering trees can provide early and abundant floral resources within a small footprint [102,103], while also providing additional non-floral resources such as nesting sites, resins and shelter [7]. Incorporating trees and other more permanent habitat features into cropping systems is key to supporting future multifunctional landscapes, both in terms of the costs of establishment and maintenance and through their role in supporting multiple ecosystems services and biodiversity [104].

### (b) Managing non-floral resources

Non-floral resources are required to support populations of bees and non-bees [7], yet manipulation studies of these resources in crop pollination contexts are infrequent, especially for non-bees [105,106]. These resources may include water bodies, manure, animal carrion, larval plant or animal host tissues, nesting materials and woody vegetation [6,7,107]. Integrating non-floral resource management into crop production may provide benefits beyond pollination, for example, through supporting non-bee crop pollinators that provide multiple ecosystem services such as nutrient recycling or biocontrol of pests [6]. These interventions may also come with risks and challenges, such as the potential for non-bees to disperse away from crops or to spread livestock pathogens [108]. Decisions to implement non-floral resource management to support non-bees will depend on evaluating both the pollinator dependency of a target crop and the pollinator efficiency of crop flower visitors. This will require the combined skills and efforts of ecologists, taxonomists and land managers in identifying which taxa are best targeted for management, their life-histories and how these can be integrated with crop and land management practices. As in efforts to enhance bee health through habitat enhancement, these interventions must be closely monitored and evaluated to ensure desired outcomes are being met.

### (c) Supplemental feeding with artificial diets

Supplemental feeding of carbohydrates or pollen substitutes is a common practice among beekeepers and an important tool in apiary management used to strengthen bee colonies and manipulate colony foraging behaviour to benefit crop production [109–111]. Besides the use of artificial diets for laboratory rearing, few studies have examined its role in the management of non-bee crop pollinators [25]. While supplemental feeding is known to support colony growth and health [109] and improve crop pollination in some circumstances [110,112], pollinator health and crop pollination outcomes have rarely been examined in tandem. Colony health outcomes can vary depending on the formulation of artificial diets [113], suggesting more research is needed into nutritional profiles and comparisons between outcomes as a result of supplemental feeding versus the provision of additional floral resources at crop sites [114]. While supplemental feeding offers the potential to mitigate nutritional deficiencies impacting pollinator health, it is an intensive management strategy which adds a substantial financial burden to beekeepers providing crop pollination services [111]. Enhancing pollinator habitat around crops together with better quality artificial feed, have potential to improve crop pollination and apicultural security.

### (d) Reducing crop pollination demand

Crops have been actively bred for greater pollen self-compatibility and parthenocarpic traits for at least 70 years [115] and this continues to be an important field of research to reduce crop pollinator dependency. More recently, biotechnology and exogenous phytohormone application have emerged as methods to create or induce parthenocarpy in crops, yet adoption of these practices will depend on the economics of production for growers and acceptance of these commodities by consumers [116]. For crops reliant on cross-pollination, pollen donor identity, spatial arrangement and phenology can impact the supply of suitable pollen in cropping systems and this has repercussions for pollinator efficiency and crop yields. Selecting suitable pollen donors and optimizing their arrangement in crop systems for pollinator-mediated cross-pollination, for example, through spatially alternating cultivar plantings, grafting or providing scattered pollen donor bouquets, may indirectly reduce crop pollinator dependency [117]. Reducing crop pollinator dependency may improve crop production by increasing yields independently of pollinator abundance, which may also translate into reduced competition between managed taxa and wild pollinators [118]. Different crop cultivars can vary in nectar and pollen quality [41] and crop domestication can alter floral chemistry with potentially negative impacts on pollinator health [119]. Breeding crop plants that have high pollinator attractiveness and offer more nutritious rewards may also alleviate some of the pressures faced by pollinators in agricultural landscapes.

### (e) New methods

Given the complexity of pollination ecology, new methods that increase our understanding of the interactions between nutritional resources, pollinator health and crop production are needed. Major technical challenges to this research include evaluating the nutritional quality of crops and other resources for specific pollinators, controlling for landscape and other variables in field studies and the designing of programmes to evaluate changes in pollinator communities and pollination over time.

Unmanned aerial vehicle mapping of vegetation [120] and harmonic radar tracking techniques to monitor pollinator movements are now available [121] and may enable high accuracy modelling of the spatial and temporal variations in pollinator activity and resource availability around crop sites. When combined with sensitive chemical analytical techniques to determine the chemical profiles of pollen and nectar [122], these technologies may enable more accurate estimation of the quality and distribution of floral resources around farms, which is essential information for field-based studies on habitat enhancement. To complement field studies, understanding how specific pollinators respond to the nutritional profiles of crops is also needed, yet collecting sufficient pollen for feeding experiments is a major challenge [75]. Incorporating live plants into enclosed feeding experiments may offer an alternative option and may also provide valuable insights into how pollinators respond to enclosed conditions, which are becoming increasingly common in crop production [25]. Portable remote monitoring tools are readily available for hives, allowing data collection in real time at low cost [123], opening the potential for broad scale studies linking environmental variables to colony dynamics. Advances in computer vision and video surveillance may facilitate field-data collection, alleviating human resource constraints to pollination fieldwork [124].

Methods such as DNA metabarcoding are becoming more accurate and are decreasing in cost. While limitations exist [125], these techniques may enable greater resolution of pollination networks and trophic interactions, for example, by identifying the origins of gut or faeces contents in adult or larvae non-bees [126]. Biomarkers are used as measures of honeybee health [13] and efforts to identity biomarkers in non-bee pollinators may be valuable in monitoring these taxa. Frameworks such as ecological stoichiometry and nutritional geometry will facilitate our understanding of these nutritional interactions and how they can be managed to best support pollinator health and crop production [82,127].

While new methods develop and expand the toolbox of research and management, we can still draw upon existing methods to better understand the mechanisms that are at play in crop pollination. Overcoming geographical and taxonomic biases in crop pollination research will facilitate our understanding of simple, yet fundamental questions that remain in regard to yield responses and insect visitation [1,128]. Greater research investment into these neglected areas will probably provide novel insights into the drivers of pollinator community assembly and reveal new pathways of pollinator management to achieve global human food security.

## 8. Conclusion

Pollinator nutrition is intricately linked to human food production through crop pollination service delivery. Enhancing crop pollinator health through nutrition will require a greater understanding of both the type and quality of nutritional resources required for specific taxa. Current initiatives aimed at increasing floral diversity around farms provide clear benefits in terms of conservation and biocontrol outcomes; however, to secure ongoing future crop pollination services, a greater understanding of the interaction between crop and non-crop floral resources is needed. While managed bees will probably remain important pollinators for many crops, cropping systems that provide the resources to support a diversity of bee and non-bee crop pollinators and other beneficial organisms will improve conservation outcomes and reduce reliance on managed bees. New research is needed to prioritize management to support the health of pollinator taxa while simultaneously ensuring pollination outcomes are being met, and negative spill-over impacts are minimized. Meeting these objectives will require greater collaboration between industry, land managers and researchers and greater investment in understanding the pollination ecology of neglected crops across different geographical regions. Tailoring habitat enhancement strategies to meet specific crop and pollinator needs and linking these to broader conservation and multifunctional landscape objectives is the next step towards achieving these goals.

## Data Availability

This article has no additional data.
